# The evaluation of biogenic silica in brackish and freshwater strains reveals links between phylogeny and silica accumulation in picocyanobacteria

**DOI:** 10.1128/aem.02527-24

**Published:** 2025-03-27

**Authors:** Anabella Aguilera, Daniel Lundin, Evangelia Charalampous, Yelena Churakova, Christian Tellgren-Roth, Sylwia Śliwińska-Wilczewska, Daniel J. Conley, Hanna Farnelid, Jarone Pinhassi

**Affiliations:** 1 Department of Biology and Environmental Science, Centre for Ecology and Evolution in Microbial Model Systems (EEMiS), Linneaus University 620860 , Kalmar, Sweden; 2 Science for Life Laboratory, Department of Immunology, Genetics and Pathology, Uppsala University 195558 , Uppsala, Sweden; 3 Mount Allison University 7017 , Sackville, New Brunswick, Canada; 4 Laboratory of Marine Plant Ecophysiology, University of Gdansk 551799 , Gdynia, Poland; 5 Department of Geology, Lund University 225265, Lund, Sweden; University of Delaware, Lewes, Delaware, USA

**Keywords:** silicon accumulation, brackish picocyanobacteria, biosilicification, *Synechococcus*

## Abstract

Through biosilicification, organisms incorporate dissolved silica (dSi) and deposit it as biogenic silica (bSi), driving the silicon (Si) cycle in aquatic systems. While Si accumulation in marine picocyanobacteria has been recently observed, its mechanisms and ecological implications remain unclear. This study investigates biosilicification in marine and brackish picocyanobacteria of the *Synechococcus* clade and two model freshwater coccoid cyanobacteria. Brackish strains showed significantly higher Si quotas when supplemented with external dSi (100 µM) compared to controls (up to 60.0 ± 7.3 amol Si.cell-1 versus 9.2 to 16.3 ± 2.9 amol Si.cell-1). Conversely, freshwater strains displayed no significant differences in Si quotas between dSi-enriched treatments and controls, emphasizing that not all phytoplanktons without an obligate Si requirement accumulate this element. The Si-accumulating marine and brackish picocyanobacteria clustered within the *Synechococcus* clade, whereas their freshwater counterparts formed a distinct sister group, suggesting a link between phylogeny and silicification. Rapid culture growth caused increased pH and led to dSi precipitation, influencing apparent dSi uptake; this was mitigated by pH control through bubbling. This phenomenon has significant implications for natural systems affected by phytoplankton blooms. In such environments, pH-induced silicon precipitation may reduce dSi availability impacting Si-dependent populations like diatoms. Our findings suggest brackish picocyanobacteria could significantly influence the Si cycle through at least two mechanisms: cellular Si accumulation and biologically induced changes in dSi concentrations.

**IMPORTANCE:**

This work provides the first evidence of biogenic silica accumulation in brackish picocyanobacteria and uncovers a link between phylogeny and biosilicification patterns. Our findings demonstrate that picocyanobacterial growth induces pH-dependent silica precipitation, which could lead to overestimations of cellular Si quotas by up to 85%. This process may drive substantial silica precipitation in highly productive freshwater and coastal marine systems, with potential effects on silica cycling and the population dynamics of Si-dependent phytoplankton. The extent of biosilicification in modern picocyanobacteria offers insights into the rock record, shedding light on the evolutionary and ecological dynamics that influence sedimentary processes and the preservation of biosilicification signatures in geological formations. Overall, this research adds to the significant impact that microorganisms lacking an obligate silica requirement may have on silica dynamics.

## INTRODUCTION

Silicon (Si) is taken up from the environment in the form of dissolved silica (dSi, orthosilicic acid) and used by several groups of eukaryotes to build skeletal structures, in a process known as biosilicification (1). Diatoms are one of the main contributors to global marine primary productivity and have long been considered the most important biosilicifiers in contemporary marine ecosystems (2). By taking up dSi to build their frustules, diatoms represent the main bridge between silica, carbon, and other biogeochemical cycles, playing a major role in carbon and biogenic silica (bSi) export to the deep oceans (2, 3). Nevertheless, in the last decade, field and laboratory studies have revealed that the marine Si cycle could be more complex than previously assumed. For example, marine picophytoplankton (prokaryotic and eukaryotic phototrophic cells < 2 µm in diameter) have been suggested to play a significant role in Si cycling as they accumulate substantial amounts of Si relative to their cell size and/or volume despite no obligate need for this nutrient (4–8). Recent estimates show that picophytoplankton represents on average ~50% of total biogenic Si (bSi) standing stocks in the oligotrophic eastern Indian Ocean and the western Pacific Ocean (9, 10) and contribute to a significant proportion of bSi in the Sargasso Sea (9%–24%) (5, 6) and the tropical South Pacific (11%–26%) (6).

Examination of cyanobacterial cells from the eastern equatorial Pacific and the Sargasso Sea demonstrated the accumulation of Si in the picocyanobacterium *Synechococcus*, with Si:P ratios comparable to those of diatoms (4, 11). These observations were corroborated by those of laboratory studies showing Si accumulation in several marine *Synechococcus* strains when exposed to increasing concentrations of dSi (4, 5). Although growth rates were not affected by dSi in external concentrations between 1 and 120 µM, Si accumulation varied significantly among strains (5), as previously seen in natural picocyanobacterial populations (4, 11). So far, no direct link has been established between this variability and either the eco-physiology or the phylogeny of the tested strains.

Biosilicification in eukaryotes has been intensively investigated. The process involves a mechanism for dSi uptake in an acidic silica deposition vesicle where controlled amorphous bSi precipitation occurs (12, 13). Molecular comparisons allow for the hypothesis that biosilicification has arisen independently in multiple different eukaryotic lineages (1, 13–17). In diatoms, several processes related to biosilicification are transcriptionally regulated by the availability of dSi (18, 19) and dSi uptake is mediated by high-affinity dSi transporters (SITs) (20). An extended family of SIT-related transporters (SIT-Ls) are involved in the uptake of dSi in non-siliceous haptophytes (16). By contrast, biosilicification has been less studied in bacteria and archaea. Recent studies reported biosilicification in heterotrophic and phototrophic bacteria (4, 21) and the presence of SIT-Ls in a few heterotrophic bacteria and *Synechococcus* strains (17). While experimental verification of SIT-L functioning is still lacking in *Synechococcus*, Si accumulation is observed in *Synechococcus* strains without SIT-Ls (4, 5, 22). The underlying cellular mechanisms leading to Si deposition remain unknown (7, 21), and research suggests that it is not bSi that accumulates in marine picocyanobacterial cells, but rather a hydrated siliceous network with Mg and/or Ca cations (23). It is worth noting that the bulk alkaline-based digestion methods widely used to analyze bSi in cells (4, 5, 8) measure it indirectly. Moreover, Si accumulation can be highly influenced by pH conditions in the culture media as inorganic Si precipitation can occur under elevated pH conditions (>8.5) (24). This calls for complementary methods to assess biosilicification and a detailed clarification of the influence of pH on Si estimates in picocyanobacteria.

While most studies documenting bSi among picocyanobacteria have been conducted in marine waters, picocyanobacteria also play an important role in primary production in brackish and freshwater systems, where diverse populations coexist and can reach high abundances (25–28). Lakes, brackish waters, and coastal embayments provide optimal circumstances for bSi production owing to their residence times (29, 30), with dSi concentrations ranging from 10 to 160 µM in brackish areas (31, 32) and 30 to 200 µM in lakes, rivers, and reservoirs (29, 33). Interestingly, elemental composition analysis of freshwater phytoplankton revealed populations of *Microcystis aeruginosa* and *Dolichospermum flosaquae* containing high amounts of bSi (34, 35), which suggests that freshwater cyanobacteria can also accumulate this nutrient. Whether biosilicification in picocyanobacteria is restricted to open oceans or also extends along the salinity gradient is currently unknown. Noteworthy, a recent study showing bSi accumulation in estuarine picoeukaryotes suggests an influence of coastal picophytoplankton on Si cycling (8). A better understanding of the distribution of bSi accumulation in picocyanobacteria in diverse aquatic habitats is needed to assess their potential significance for the global Si cycle and other biogeochemical cycles.

In this study, we analyzed the current knowledge on the distribution of biosilicification in brackish picocyanobacteria belonging to *Synechococcus*/*Prochlorococcus* clade (Syn/Pro clade, hereafter) along with two divergent freshwater coccoid strains: *Synechococcus elongatus* PCC 7942, an often-used model for freshwater *Synechococcus,* and *Synechocystis* PCC 6803, a model organism for research in phototrophic bacteria. We combined molecular, biochemical, and bioinformatic approaches to provide insights into the poorly understood mechanisms of biosilicification in picocyanobacteria. Moreover, we quantified Si precipitation induced by increases in the pH due to picocyanobacterial growth and discussed its potential role in biological control of dSi concentrations and ecological implications in natural systems.

## MATERIALS AND METHODS

### Picocyanobacterial strains and culture conditions

A total of 37 picocyanobacterial strains were used in this study, including marine and freshwater reference strains and newly isolated brackish strains, diverse in terms of pigment composition. All the strains were screened for the presence of SIT-Ls using specific primers, and we explored their placement within known picocyanobacterial phylogenetic clades (Table S1). Eleven strains were tested for silica accumulation (Table 1).

**TABLE 1:** Strains used in this study for silica accumulation

Strain	Origin	Isolation site	Growth media	Obtained from
*Synechococcus* sp. WH 5701	Marine, coastal	Atlantic Ocean, Long Island	L1 media in artificial seawater (35 PSU)	Roscoff Culture Collection (RCC)
*Synechococcus* sp. BA 120	Estuarine	Gulf of Gdańsk (southern Baltic Sea)	L1 media in artificial seawater (7 PSU)	Culture Collection of Baltic Algae (CCBA)
*Synechococcus* sp. BA 124	Estuarine	Gulf of Gdańsk (southern Baltic Sea)	L1 media in artificial seawater (7 PSU)	Culture Collection of Baltic Algae (CCBA)
*Synechococcus* sp. BA 132	Estuarine	Gulf of Gdańsk (southern Baltic Sea)	L1 media in artificial seawater (7 PSU)	Culture Collection of Baltic Algae (CCBA)
*Synechococcus* sp. KAC 102	Estuarine	Baltic Sea Proper	L1 media in artificial seawater (7 PSU)	Kalmar Algal Collection (KAC)
*Synechococcus* sp. KAC 105	Estuarine	Baltic Sea Proper	L1 media in artificial seawater (7 PSU)	Kalmar Algal Collection (KAC)
*Synechococcus* sp. KAC 106	Estuarine	Baltic Sea Proper	L1 media in artificial seawater (7 PSU)	Kalmar Algal Collection (KAC)
*Synechococcus* sp. KAC 108	Estuarine	Baltic Sea Proper	L1 media in artificial seawater (7 PSU)	Kalmar Algal Collection (KAC)
*Synechococcus* sp. KAC 114	Estuarine	Baltic Sea Proper	L1 media in artificial seawater (7 PSU)	Kalmar Algal Collection (KAC)
*Synechococcus elongatus* PCC 7942	Freshwater	California, USA	BG11	Pasteur Culture Collection of Cyanobacteria (PCC)
*Synechocystis* sp. PCC 6803	Freshwater	California, USA	BG11	Pasteur Culture Collection of Cyanobacteria (PCC)

Cultures were grown in L1 media (36) in artificial seawater (7 PSU for the Baltic Strains and 35 PSU for the marine strains) and prepared with artificial sea salt (Sigma-Aldrich). Freshwater strains were grown in BG11 media (37). Cultures were grown in media without dSi addition and kept in polycarbonate bottles at 18°C, at 100 µmol photons m^−2^ s^−1^ irradiance on a 12 hour light:12 hour dark photocycle. Media and stock solutions were prepared and stored in polycarbonate bottles to reduce additional sources of dSi (e.g., dSi is produced in alkaline solutions preserved in glass containers (21). Notably, dSi-free media has been proven to be cause difficulties to obtain given trace amounts of dSi in culture media components and/or tap water (5, 21). Accordingly, in our experiments, the dSi concentrations in the controls before inoculations were in the low µM range: ~1.4 µM in L1 7 PSU, ~2.2 µM L1 35 PSU, and ~4.4 µM in BG11.

### Cellular bSi accumulation experiments

Eleven picocyanobacterial strains were tested for Si accumulation (Table 1). *Synechococcus* sp. WH 5701 was used as a positive control as Si accumulation has been previously reported for this marine strain (4, 5). Eight strains from the Baltic Sea which have been recently characterized physiologically were selected as brackish strains (38, 39). *Synechococcus elongatus* PCC 7942, an often-used model for freshwater *Synechococcus,* and *Synechocystis* sp. PCC 6803, a model organism for research in prokaryotic phototrophs, were used as freshwater representatives.

Elevated pH conditions (> 8.5), commonly found in batch cultures, can induce inorganic Si precipitation in the form of sepiolite (Mg_2_Si_3_O_8_), compromising cellular Si measurements (23, 24). We first assayed two treatments to regulate pH and evaluated their impact on Si accumulation in two brackish strains (BA 120 and BA 124) and one marine (WH5701): (i) bubbling cultures with pre-sterilized ambient air (passage through a 0.2-µm filter before entering each culture bottle); (ii) growing cultures with agitation (orbital shaking 100 rpm) and buffered with (4-(2-hydroxyethyl) piperazine-1-ethanesulfonic acid (HEPES) pH 7.5 (Sigma-Aldrich) at two concentrations (5 mM and 10 mM). Culture media were set to pH 7.5 before inoculation, and pH was closely monitored daily during the experiments. After assessing the two treatments to regulate pH, we chose to bubble the cultures to proceed with the experiments.

To evaluate bSi accumulation, cells of cultures in the early exponential phase were used to inoculate culture media supplemented with 100 µM dSi. This concentration does not affect the growth of eukaryotic and prokaryotic picophytoplankton without Si requirements (5, 8, 22) and would be within the range where dSi uptake can be mediated by transporters (5). L1 and BG11 media without dSi addition were used as controls. The physiological stability of the cell lines was maintained by acclimating them to the experiment conditions for 15–18 days. Cell lines in the exponential phase were transferred to the fresh culture medium at least three times before the experiments.

Growth was monitored daily by optical density (OD 750_nm_) using a multimode microplate reader (FLUOstar Omega, BMG LABTECH) as this parameter showed a strong positive linear correlation with the cell concentration determined by flow cytometry and cell counts. When cultures reached the mid-exponential phase, aliquots were taken for dSi, bSi, and cell counts (see below). Additional samples were taken for strain WH 5701 (marine) and KAC 102 (brackish) to evaluate the effect of dSi addition using the whole transcriptomic analysis.

To estimate the cell size (µm), the diameters of 30 cells were measured under the epifluorescence microscope (Olympus BX50) at 1,000 x magnification. Cell sizes were used to estimate the average biovolume (µm^3^) using geometric shapes (sphere and spheroid) (40).

We also investigated the extent of inorganic precipitation that can be produced in batch cultures of picocyanobacteria when pH is not regulated. Cells at the early exponential phase growing without dSi addition were used to inoculate culture media supplemented with 100 µM dSi. For this experiment, triplicate cultures were grown in an orbital shaker (100 rpm) under the temperature and light conditions described above. Daily sampling was done to monitor growth (by optical density, OD 750_nm_), pH evolution, and dSi. When cultures reached the mid-exponential phase, aliquots were taken for cellular bSi quotas and cell counts.

### dSi, bSi, and dry weight determination

For dSi, cell suspensions (5 mL) were filtered through a 33-mm-diameter 0.22 µm pore-size Millex-GP filter (Millipore, Ireland) and stored at −20°C to determine the concentration of dSi using a silicate molybdate ascorbate assay following the method outlined by Hansen and Koroleff (41). To determine bSi quotas, cell suspensions (20–30 mL) were filtered onto 47-mm-diameter 0.2 µm pore-size Nuclepore Track-Etch filters (Whatman, United Kingdom). The filters were stored in Teflon tubes at −20°C until digestion in 0.2 M NaOH at 95°C for 1 hour (5, 42). Si was transformed into dSi and measured with a UV-1600PC spectrophotometer (Shimadzu, Japan) at a wavelength of 810 nm using 50-mm cuvettes. Culture aliquots (1 mL) were fixed with Lugol’s solution for cell counts under the microscope using a Neubauer chamber. This approach was combined with flow cytometry to avoid errors in cell counting as some of the strains used in the present study form aggregates with a variable number of cells (39). Cellular silicon quotas were calculated as total bSi divided by cell abundance. It is worth mentioning that bSi determination following this approach includes Si from both living cells and detrital material.

For dry weight determinations, cell suspensions (~ 500 mL) were harvested in the mid-exponential phase (OD_750 nm_≈ 0.3) at 6,000 × *g* for 20 minutes at 4°C and freeze-dried (VirTis BenchTop Pro) at 0.13 millibar until complete dryness.

### DNA extractions

Samples for total genomic DNA were collected by harvesting 10 mL of each culture and centrifuging for 8 minutes at 8,000 x *g*. DNA was extracted using the FastDNA SPIN Kit for soil (MP Biomedicals) with Lysing Matrix E tubes (MP Biomedicals) following the manufacturer’s instructions with the addition of an incubation with proteinase-K (1% final concentration) at 55°C for 1 hour directly after homogenization with a FastPrep-24 5G bead beating grinder and lysis system. DNA concentration was measured using an Invitrogen Qubit 2.0 fluorometer (Thermo Fisher Scientific Inc.), and purity was assessed using a Thermo Scientific NanoDrop 2000 spectrophotometer (Thermo Fisher Scientific Inc.). The same procedure was followed to extract DNA for whole-genome sequencing, with the exception that the homogenization was performed with a Vortex mixer prior to incubation with proteinase-K. Samples were stored at −20°C until sequencing at the Swedish National Genome Infrastructure, SciLifeLab, Stockholm.

### RNA extraction and sequencing

The responses to dSi addition were assessed by whole transcriptomic analysis in two picocyanobacterial strains: WH 5701 (marine) and KAC 102 (brackish). Samples for total RNA extraction were collected at the exponential phase by harvesting 30 mL of cultures grown in media with no Si addition (control) and dSi-enriched media (+100 µM), at the exponential phase. After centrifuging for 12 minutes at 8,000 x g, cell pellets were snap-frozen with liquid nitrogen and stored at −80°C until further use. Samples were thawed on ice, and total RNA was extracted using the RNeasy Mini Kit (Qiagen) following a protocol adapted from (43). Briefly, cell pellets were resuspended in a lysis mix (1 mL of buffer RLT plus 10 µL β-mercaptoethanol) and added to Lysing Matrix E tubes (MP Biomedical). After three runs of homogenization with a FastPrep-24 5G bead beating grinder and lysis system (6.0 m s^−1^ for 30 seconds), samples were centrifuged for 5 minutes at 5,000 x *g* at 4°C. Each sample supernatant was then transferred into tubes containing an equal volume of 70% ethanol, which were well mixed by pipetting multiple times. The total RNA was eluted two times with 30 µL of RNase-free water (preheated at 50°C). DNA was removed with the TURBO DNA-free Kit (ThermoFisher Scientific), and ribosomal RNA was depleted using the RiboMinus Transcriptome Isolation Kit and RiboMinus Concentration Module (ThermoFisher Scientific). RNA integrity was assessed with TapeStation (Agilent), and the quality was measured using a NanoDrop 2000 spectrophotometer (Thermo Fisher Scientific Inc.), and the concentration was quantified with a Qubit 2.0 fluorometer (Thermo Fisher Scientific Inc.). RNA samples were kept at −80°C until sequencing at Eurofins Genomics on Illumina NovaSeq 6000 S4, PE 2 × 150 bp. Transcript sequences are available at the European Nucleotide Archive (ENA) under the accession number PRJEB76927.

Reads were mapped to the reference genome with a pre-release version of nf-core/magmap (revision 6518a0dc1b; https://github.com/erikrikarddaniel/magmap). The pipeline uses Trimgalore (v. 0.6.7; https://github.com/FelixKrueger/TrimGalore) and Cutadapt (44) to cut adapters from sequences and quality trim them. Trimmed quality reads are subsequently mapped to the respective reference genomes (strains WH5701 and KAC 102) with BBMap (v. 38.92; https://sourceforge.net/projects/bbmap/), and features are quantified with featureCounts from the Subread package v. 2.0.1 (45). The levels of expression of genes of interest were quantified as transcripts per million (TPM), i.e., gene length-adjusted relative abundance of transcripts of interest among the total population of sequenced transcripts. NMDS and Hellinger PCA analyses were performed to assess the differences between Si-treated samples (culture media supplemented with 100 µM dSi) and controls (no dSi addition) using Vegan v. 2.6.4 (46) and Tidyverse v. 2.0.0 (47) packages in R v. 4.2.1 (48). A test for differentially abundant expression of genes was performed with ALDEx2 v. 1.30.0 (49).

### Genome sequencing and annotation

One microgram of genomic DNA from strain KAC 102 was used to create a SMRTbell sequencing library according to the manufacturer’s instructions (Pacific Biosciences, Menlo Park, CA, USA) sequenced on a PacBio Sequel II instrument (Pacific Biosciences, Menlo Park, CA, USA) with 30 hours movie time. The sequencing yielded ~320 Mb of sequence data. The genome was assembled using Flye v2.8.3 (v. 2.8.3 (50), hifiasm-meta (51), and hifiasm (v. 0.16.1 (52) with default parameters, and pbmm2 was used for mapping and selecting the best contigs from each assembler for the final result. The resulting assembly was analyzed with checkM (v.1.1.3 (53) to assess the completeness and functionally annotated with Prokka (v.1.14.6 (54). We ran GTDB-Tk (v. 2.1.0 (55) using GTDB version R07RS207 (56) to establish the taxonomy of the strain (GTDB species “CBW1002 sp015840915” in the Cyanobiaceae family). The genome from *Synechococcus* sp. WH5701 was downloaded in assembly format from the NCBI (accession GCF_000153045.1) and annotated as described for KAC 102. The genome of KAC 102 is available at the European Nucleotide Archive under the accession number GCA_963920495.

### Phylogenetic analysis

We explored the phylogenetic placement of brackish and freshwater strains used in this study (*n* = 37; Table S1) and their relationship with *Synechococcus* strains previously reported to accumulate Si (5) and the strains harboring SIT-Ls (17). A fragment of the 16S rRNA gene (approximate size 1,400 pb) was amplified with universal primers 27F and 1492R (Table S2). The amplification reactions were carried out as previously described for the SIT-Ls gene. The cycling conditions were as follows: initial denaturation at 98°C for 5 minutes, followed by 30 cycles at 98°C for 40 seconds, 55°C for 40 seconds, extension at 72°C for 1 minute, and a final extension step at 72°C for 10 minutes. PCR products were sent for Sanger sequencing (Macrogen Europe, Amsterdam, the Netherlands). 16S rRNA gene sequences were aligned with MAFFT v. 7.5 using the G-INS-I algorithm (with default parameters) (57). Phylogenetic calculations were run employing maximum likelihood analysis (58, 59) and Bayesian inference in MrBayes v3.2.7a. Maximum likelihood phylogenies were constructed using IQ-TREE v. 1.6.12 with default parameters, using a GTR + F + I + I + R3 model determined by ModelFinder (60). Bootstrap values were calculated with 1,000 replicates (58). Bayesian inference phylogenies were constructed using MrBayes v3.2.7a (61) under the GTR + I + gamma model (lset nst = 6 rates = invgamma). Two runs of eight Markov chains were executed for 20,000 generations with default settings for the analysis command (mcmc; one cold chain and three heated chains), and the final average standard deviation of split frequencies was lower than 0.01.

### Data analysis

Experiments testing Si accumulation were conducted with three or four biological replicates, and results are presented as the mean value (± standard deviation, SD). At least three independent experiments were performed. Specific growth rates were calculated using optical density (OD 750_nm_) in the exponential phase following the equation:


$$\mu =\frac{\ln\left(\frac{OD_{t_{f}}}{OD_{t_{i}}}\right)}{t_{f}-t_{i}}$$

where *t*_*f*_ and *t*_*i*_ represent the final and initial days of the exponential phase, respectively.

Differences in cellular bSi quotas and growth rates obtained under different treatments and different strains were analyzed with one and two-way ANOVA, respectively. The Shapiro–Wilk test was used to test the normal distribution of the residuals, and data were square-root-transformed when necessary. When significant differences (*P* < 0.05) were found, the Tukey *post-hoc* test was used for multiple comparisons within groups. Plots were created in GraphPad Prism Version 9.3.1 for MacOS. Statistical analyses were performed using car and lsmeans packages in R (48).

### Searches for dSi transporters and proteins related to Si deposition in picocyanobacteria

We first searched for dSi transporters in public databases. The GTDB database was searched for proteins containing conserved sequence motifs for silicon transporters as defined by Pfam HMM profile PF03842 using the hmmsearch, part of the HMMER package v. 3.3.2 (62). Searches were also performed in the Baltic Sea Reference Metagenome (BARM), a reference catalog for the Baltic Sea gathering 2.6 billion metagenomic reads from 81 water samples, spanning both spatial and temporal dimensions, which contains 6.8 million genes annotated for function and taxonomy (63).

Using the nf-core/phyloplace workflow v. 1.0 (64), hits were placed onto a previously published amino acid-based reference tree (17). The workflow uses Nextflow (65) and is part of the nf-core collaboration (66). It uses HMMER v. 3.3.2 (62) to align query sequences to a reference alignment and subsequently places them in a reference phylogeny with EPA-NG v. 0.3.8 (67). Finally, a phylogenetic tree containing reference and query sequences is produced with Gappa v. 0.8.0 (68).

Based on a sequence found in BARM, which exhibited the predicted motifs described for bacterial SIT-Ls (17) and assigned to *Synechococcus* sp., we designed two sets of primers (Table S2) that were used to screen for SITs-L in genomic DNA extracted from 34 selected picocyanobacterial strains isolated from the Baltic Sea (Table S1). The amplification reactions were carried out in a 25 µL final volume containing 10 ng of template DNA, 0.5 mM of each primer, and commercial PCR mix (Phusion High-Fidelity PCR Master Mix, Thermo Scientific) on a T100 Thermal Cycler (BIO RAD, USA). The cycling conditions were as follows: initial denaturation at 94°C for 5 minutes, followed by 35 cycles at 94°C for 40 seconds, 60°C for 40 seconds, extension at 72°C for 30 minutes, and a final extension step at 72°C for 10 minutes. A double-stranded laboratory-synthesized DNA fragment (GeneStrand, Eurofins Genomics) with the SIT-L sequence found in the BARM database was used as a positive control in the PCRs.

As an attempt to further understand the molecular mechanism of biosilicification in picocyanobacteria, we screened the GTDB database for silica-related genes and proteins related to silica deposition using BLASTn (69). Genes and proteins previously described in eukaryotic biosilicifiers were used as queries (Table S1).

## RESULTS

### Effect of pH regulation on picocyanobacterial growth and bSi quotas

**Fig 1:**
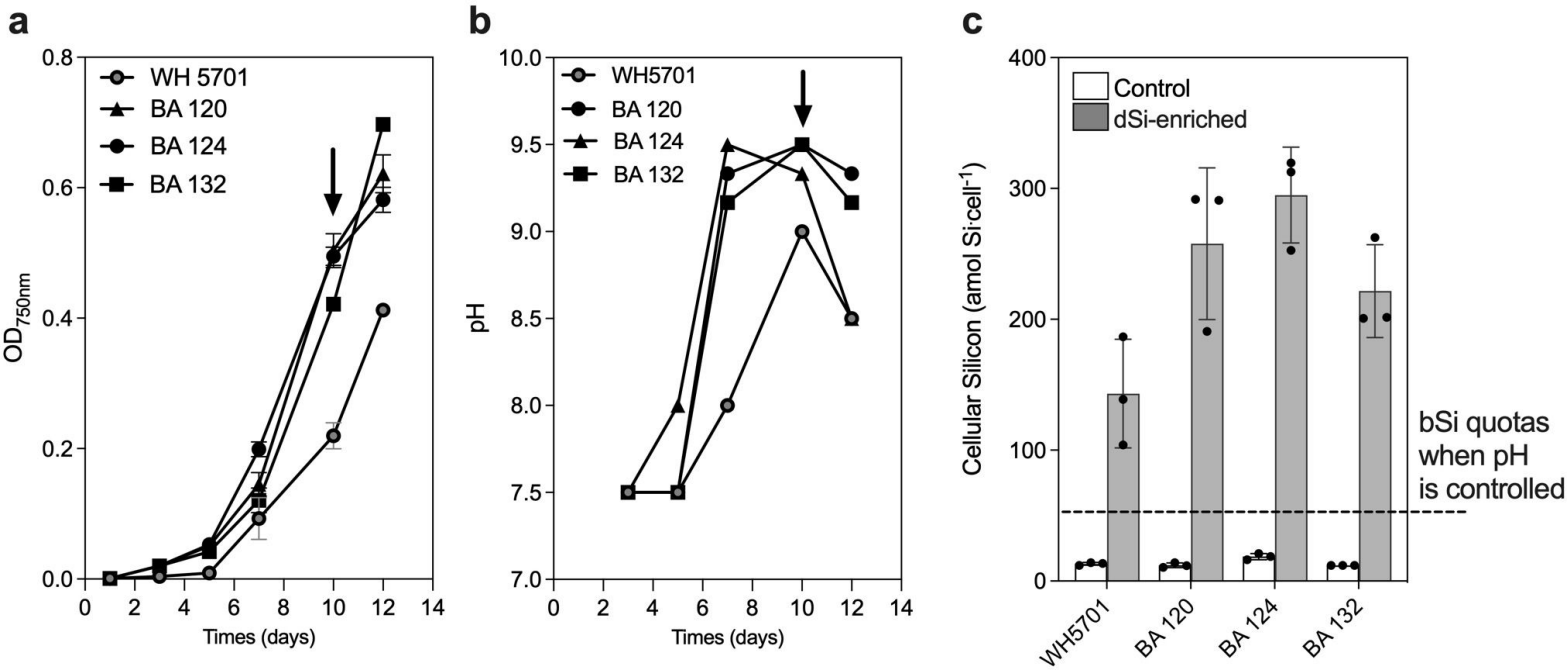
Growth curves, pH, and cellular silicon content in batch cultures of a marine (WH 5701) and three brackish (BA 120, BA 124, and BA 132) picocyanobacteria. (a) Growth curves of picocyanobacteria cultured in dSi-enriched media (+100 µM); (b) pH evolution in cultures of picocyanobacteria cultured in dSi-enriched media; (c) cellular silicon accumulation in control conditions (without added dSi) and in dSi-enriched media. The arrows indicate the time point at which the samples were collected for bSi analysis.

**Fig 2:**
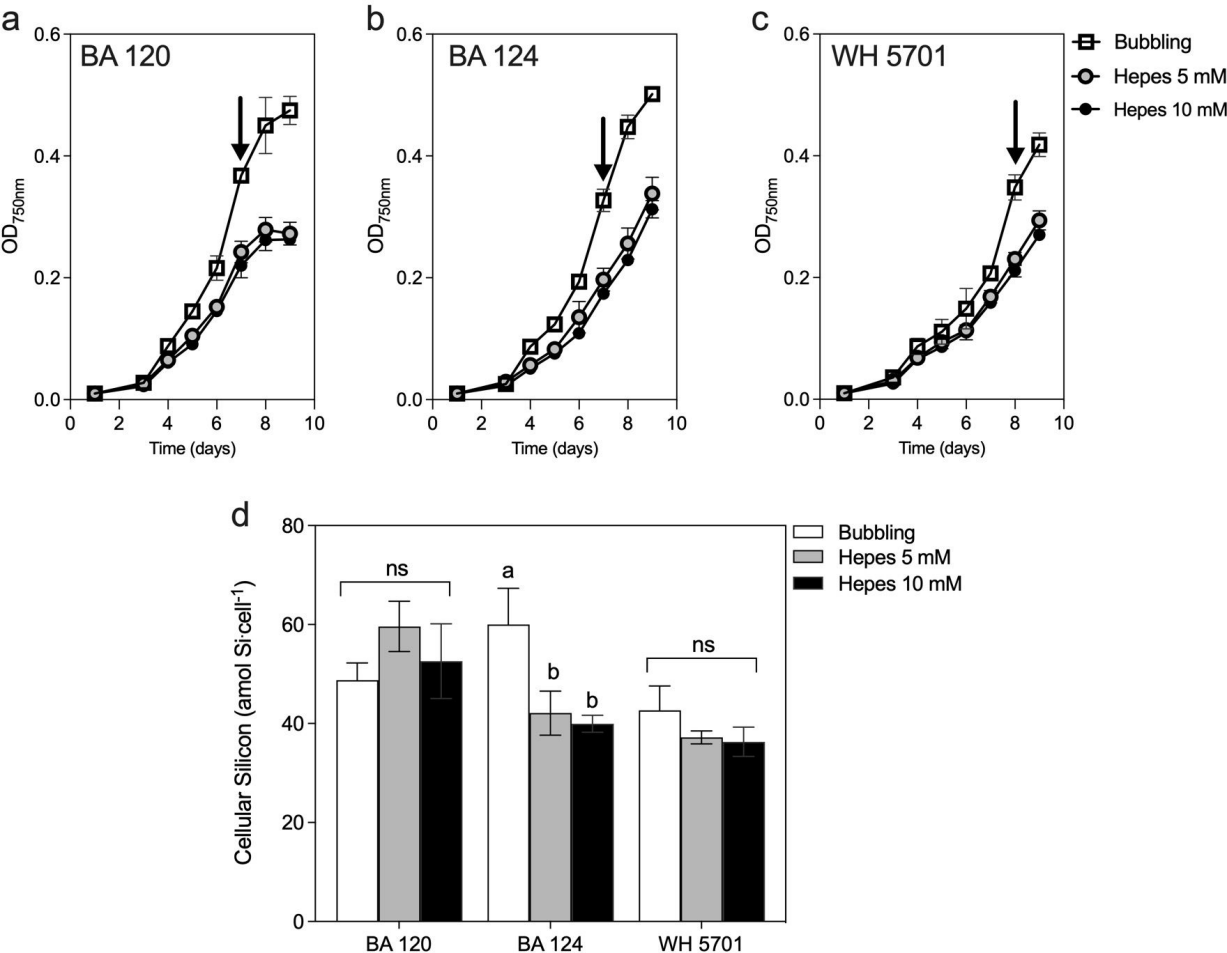
Growth and cellular silicon content in picocyanobacterial strains in bubbled or cultures buffered with HEPES. Growth curves of brackish strains BA 120 (a), BA 124 (b), and marine strain WH 5701 (c); (d) cellular silicon accumulation in strains cultured in dSi-enriched media (+100 µM) in bubble cultures or buffered with HEPES pH 7.5 5 mM or 10 mM. ns, not significant; letters a and b denote statistical differences (one-way ANOVA, *P* < 0.05; Tukey *post-hoc P* < 0.05). The arrows indicate the time point at which samples were collected for bSi analysis.

Although pH is a critical factor in regulating the solubility of silicates, i.e., the magnesium silicate sepiolite can form and precipitate at pH >8.6 (24) and interfere with the determination of cellular bSi quotas, there are few actual measurements of this effect in the literature, and we are not aware of any for picocyanobacteria. We first examined dSi precipitation induced by brackish picocyanobacteria BA 124 growing in batch cultures. In this strain, the increase in pH due to active photosynthesis was followed by a pronounced decrease in the dSi concentration in the medium from ~102 µM to ~25 µM, and dSi concentrations decreased sharply when the pH in the media surpassed 9 (Fig. S1). This result is consistent with protocols for magnesium-induced co-precipitation of Si to concentrate Si from marine waters (70, 71). Indeed, when growing BA 120, BA 124, and BA 132 in batch cultures, the pH of the media reached 9–9.5 due to the active photosynthesis after 6 to 8 days of growth (Fig. 1a and b). The media alkalinization led to average cellular Si quotas between 150 and 300 amol Si cell^−1^ in all the strains growing in dSi-enriched media (Fig. 1c). This corresponds to Si quotas threefold to sevenfold higher compared to the ones obtained when pH conditions were controlled (see below, Fig. 2).

We next evaluated two ways to regulate the pH, by determining the effect of bubbling with air and chemical buffering with HEPES on the growth and Si quotas in one marine (WH5701) and two brackish strains (BA 120 and BA 124). Bubbling and buffering maintained the pH around 8.5 and led to significant differences in growth rates (one-way ANOVA, *P* < 0.05; Table S4). While the two concentrations of HEPES applied (5 and 10 mM) led to similar growth rates, growth was slightly hampered when cultures were buffered compared to bubbled (Fig. 2a through c). In the WH 5701 and BA 120 cultures, cellular Si quotas were similar when buffering and bubbling (one-way ANOVA, *P* > 0.05). However, a significantly higher cellular Si quota was found in BA 124 when growing with bubbling compared to when buffered with HEPES (one-way ANOVA, F = 14.31; *P* = 0.005) (Fig. 2d). Changes in cellular Si quotas as a consequence of a reduced growth rate could not be excluded; therefore, cultures were bubbled in subsequent experiments.

### Effects of added dSi on bSi quota in bubbled cultures

**Fig 3:**
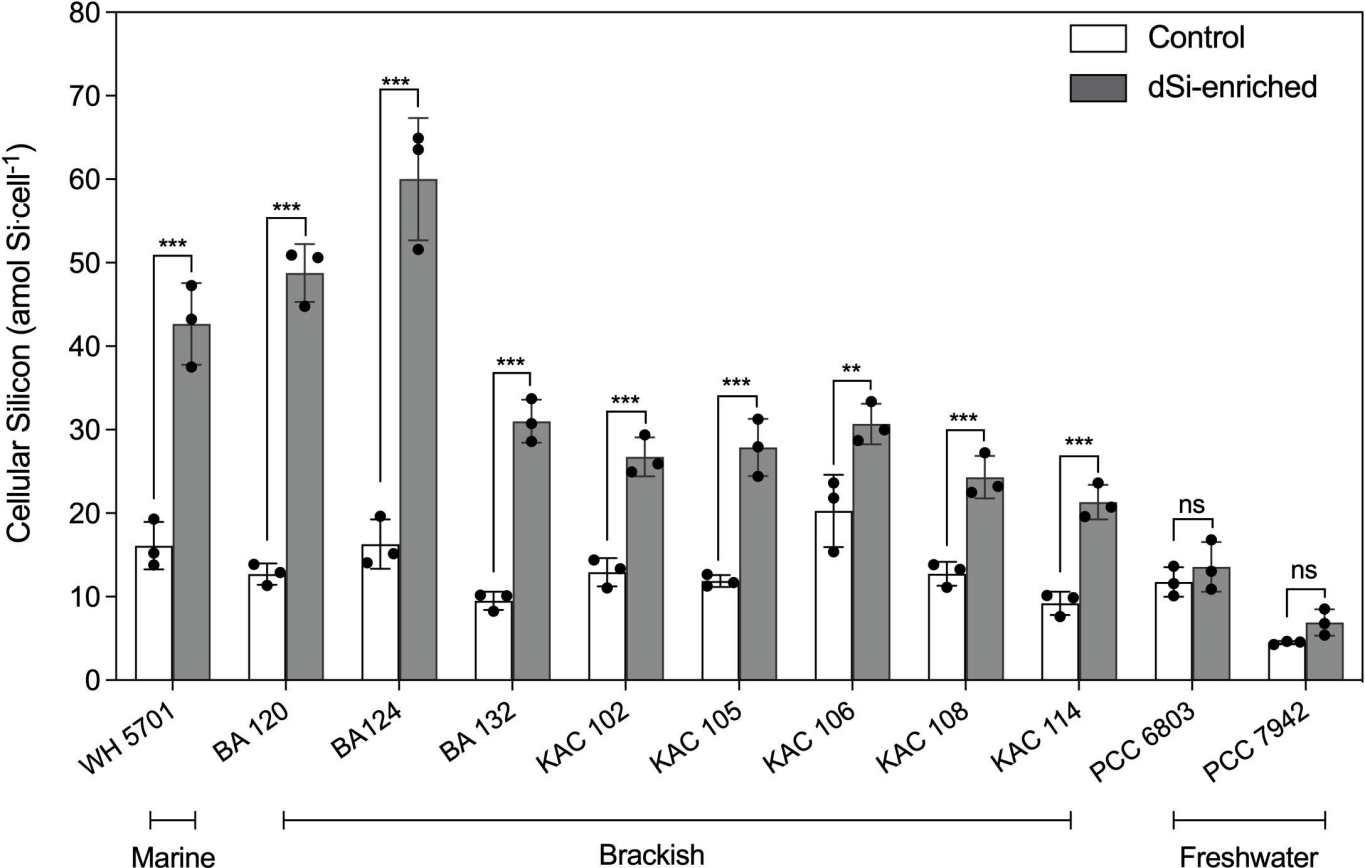
Cellular silicon content in marine, brackish, and freshwater strains. Strains were cultured in control conditions (without added dSi) and in dSi-enriched media (+100 μM). Asterisk(s) (*) indicate significant *P* values (one-way ANOVA, *P ≤ 0.05, **P ≤ 0.01, and ***P ≤ 0.001).

To obtain a broader view of the Si accumulation abilities of picocyanobacteria, we analyzed eleven strains from marine, brackish, and freshwater environments growing in bubbled cultures (Table 1). The growth curves of all picocyanobacterial strains were similar when grown with and without dSi addition (Fig. S2). Cellular Si quotas in dSi-enriched cultures were consistently higher in the marine and brackish strains compared to the controls (culture media without dSi addition) (Fig. 3). The highest quotas were found when cultures were in the middle exponential phase and decreased when cells entered the late exponential and stationary phases (data not shown). Brackish strain BA 124 had the highest average values of cellular Si quotas (60.0 ± 7.3 amol Si.cell^−1^), followed by BA 120 (48.8 ± 3.5 amol Si.cell^−1^) and the marine strain WH 5701 (42.7 ± 4.9 amol Si.cell^−1^). Average values for the other brackish strains ranged between 21 and 30 amol Si cell^−1^. In the controls, cells contained measurable cellular Si quotas, leading to background values ranging from 9.2 ± 1.4 amol Si.cell^−1^ to 16.3 ± 2.9 amol Si.cell^−1^. The freshwater strains (PCC 6803 and PCC 7942) showed no differences in cellular Si quotas between Si-treated cultures compared to the controls. Strain PCC 7942 had the lowest average values of Si in both control (4.5 ± 0.2 amol Si.cell^−1^) and dSi-enriched media conditions (6.9 ± 1.6 amol Si.cell^−1^) (Fig. 3).

Overall, we found no relationship between cell size, biovolume, and bulk dry weight measurements and cellular Si accumulation. Bulk dry weight measurements ranged between 1.2 and 1.4 µg cell^−1^ in the marine strain and between 0.3 and 0.7 µg cell^−1^ in the brackish strains (Fig. S3).

### Effects of silica addition on picocyanobacterial gene expression

Using whole-genome transcriptome analysis, we examined whether we could detect and quantify changes in gene expression in cultures with dSi addition (100 µM) compared to controls in the marine strain WH 5701 and the brackish strain KAC 102. After 8 days of growth in dSi-enriched media, cells accumulated Si (42. 7 ± 4.9 amol Si cell^−1^ in WH 5701 and 26.8 ± 2.3 amol Si cell^−1^ in KAC 102; <15 amol Si cell^−1^ in the controls). Neither nMDS nor PCA analyses on the gene expression data showed any separation of clustering between samples with or without dSi for the two strains (Fig. S4). Statistical analysis of differentially abundant genes using ALDEx showed no difference at a significance threshold of 0.05 (data not shown).

### Phylogeny of picocyanobacteria accumulating silica

**Fig 4:**
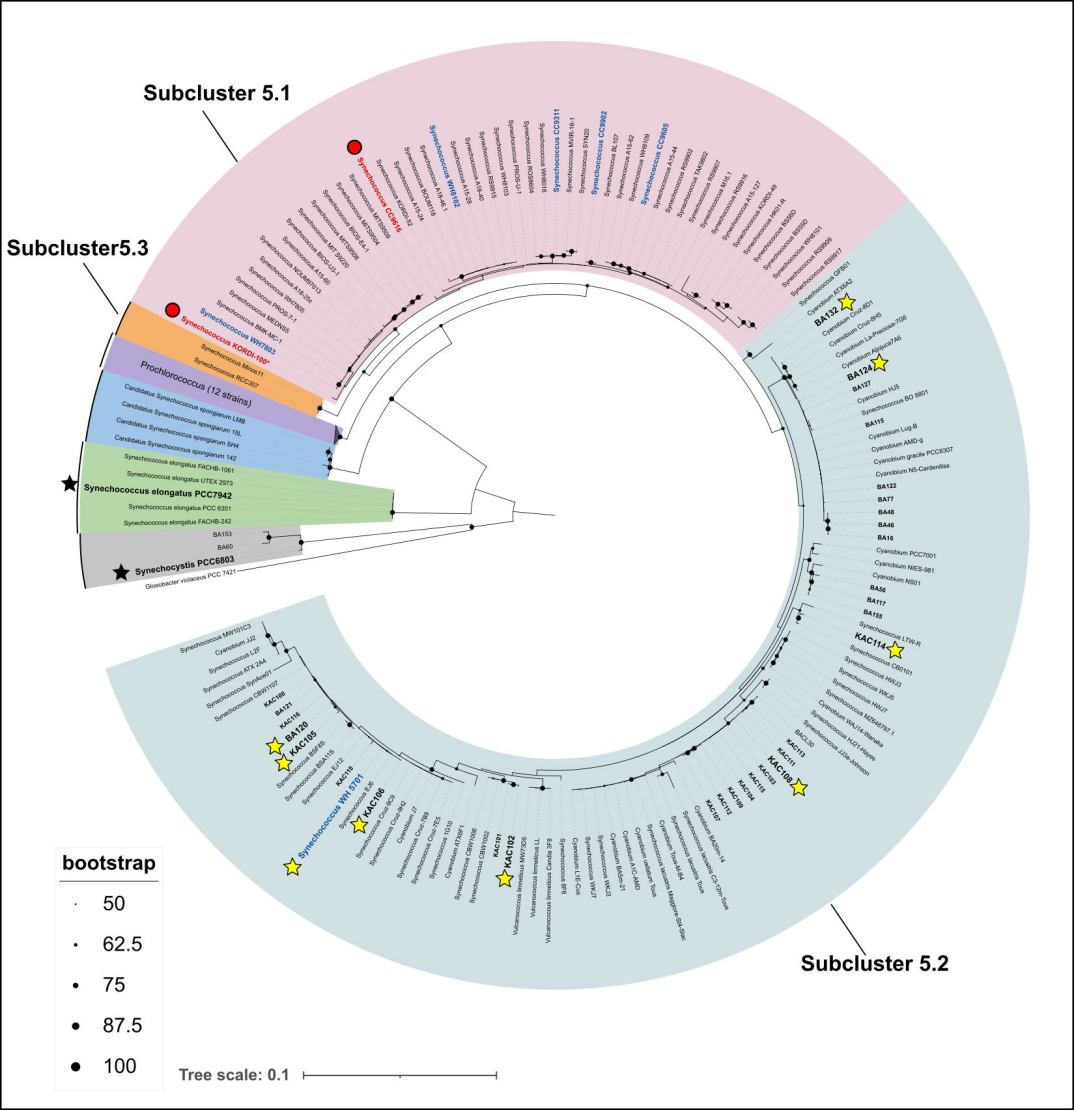
Phylogenetic position of picocyanobacterial strains used in this study and other strains shown to accumulate Si. Red circles indicate strains harboring SIT-Ls (17), and strains in blue were previously shown to accumulate Si (5). Stars indicate strains tested for Si accumulation in this study: yellow stars indicate strains that accumulated Si, and black stars indicate those that did not accumulate Si. All BA strains and KAC strains (in bold) were screened for SIT-Ls. Maximum likelihood phylogenies were constructed using a GTR + F + I + I + R3 model determined by ModelFinder (60). Bootstrap values were calculated with 1,000 replicates (58).

Phylogenetic reconstructions using the 16S rRNA gene (amplicon average size 1,385 bp) indicated that strains accumulating Si fell within the Syn/Pro clade with strong statistical support when employing maximum likelihood and Bayesian inference (Fig. 4; Fig. S5). Specifically, the brackish strains fell within sub-cluster 5.2 together with marine *Synechococcus* WH 5701 and other brackish and some freshwater strains. Marine *Synechococcus* strains known to accumulate Si (i.e., WH 8102, WH 7803, CC 9311, CC 9902, and CC 9605) fell in sub-cluster 5.1 together with the strains previously reported to harbor SIT-Ls (CC 9616 and Kordi-100). The freshwater model picocyanobacteria *Synechococcus elongatus* PCC 7942 and *Synechocystis* PCC 6803, which did not accumulate Si (see below), formed a sister group to the Syn/Pro clade.

### Bioinformatic searches and phylogenetic analysis of silicic acid transporters and silica deposition proteins

To identify potential Si-related genes in picocyanobacteria, we first searched genomes deposited in public databases for SIT-L transporters. This identified nine novel bacterial SIT-L sequences: eight among genomes in the GTDB database (release 07-RS207) and one in the BARM database (63). Within GTDB, two sequences were assigned to picocyanobacteria (an SAG assigned to *Prochlorococcus* AG-412-IO5 from the North Atlantic Ocean and an MAG assigned to *Cyanobium* sp. NAT70 from the South Atlantic Ocean), four to Proteobacteria (two *Methylomarinum* sp. MAGs, one MAG of *Arenicella* UBA7415, and a strain of *Sinobacterium caligoides*), two sequences from Bacteroidota MAGs (*Labilibaculum* sp. and *Prevotella* sp.), and one Cloacimonadota MAG sequence. The sequence found in the BARM database was taxonomically assigned to *Synechococcus* sp. (accession number k99_969740_4). The three novel sequences from Picocyanobacteria exhibited high amino acid sequence identity (45.6%–70.7%) to SIT-Ls previously identified in *Synechococcus* and contained the five predicted transmembrane domains and one EGXQ–GRQ motif pair (Fig. S6).

Using phylogenetic placement, the novel SIT-L sequences found in the GTDB (*n* = 8) and BARM (*n* = 1) data sets were placed onto a previously published phylogenetic tree of SIT and SIT-L sequences. The sequences found in this study fell within the bacterial SIT-L branch (17) (Fig. S7). Within this branch, the three novel picocyanobacterial sequences were clustered with previously annotated SIT-L sequences assigned to *Synechococcus* sp. and two contigs present in the bacterial size fractions of the Tara Oceans environmental metagenomic data sets (17). The novel SIT-Ls assigned to heterotrophic bacteria (*n* = 6) clustered with SIT-L sequences assigned to *Rhodococcus opacus* (Actinobacteria) (Fig. S7).

PCR screening of genomic DNA from 34 picocyanobacterial strains isolated from the Baltic Sea for the presence of SIT-Ls using combinations of two pairs of primers gave no positive results (PCR products only detected for the positive control of synthetic SIT-L DNA; data not shown).

We next searched for other proteins involved in dSi uptake in known eukaryotic biosilicifiers in picocyanobacterial genomes deposited in the GTDB. Our search for Lsi2 genes, coding for transmembrane transporters mediating Si efflux in plant cells (72), revealed Lsi2-like homologs in *Zea mays* (NCBI accession no. ONL92684) and in two *Synechococcus elongatus* genomes in GTDB (strains PCC 11801 and PCC 11802), which were annotated as putative transporters. Also, homologs of the Lsi2-like gene in *Thalassiosira pseudonana* (NCBI accession no. XP_002287839) were found in eight genomes assigned to *Synechococcus elongatus* in GTDB (annotated as putative transporters).

Searches of picocyanobacterial genomes deposited in the GTDB for proteins involved in bSi deposition in known eukaryotic biosilicifiers (e.g., silaffins, pleuralins, and frustulins in diatoms; silicateins and glassins in sponges; Table S3) yielded no positive results.

## DISCUSSION

The discovery of natural populations of picocyanobacteria containing significant amounts of Si revealed a previously unrecognized influence of this group on the marine Si cycle (4), especially in oligotrophic areas where this group is highly abundant and their biomass is significantly higher than those of diatoms (7). Our study expands the record of silicifying picocyanobacteria from marine *Synechococcus* to brackish strains and shows that dSi concentrations up to 100 µM do not affect growth, as reported for marine *Synechococcus* (5) and brackish and marine picoeukaryotes (8). Altogether, our findings point toward a scenario in which brackish picocyanobacteria could play a significant role in the Si cycle through at least two mechanisms influencing dSi availability: cellular Si accumulation and biologically induced dSi precipitation.

### Extending the findings to brackish environments; not all biological entities take up dSi and the first report of negative control

In the last decade, evidence has been gathered on bSi accumulation in major phytoplankton lineages without strict requirements for this nutrient. bSi accumulation has been reported in chlorophytes (*Chlamydomonas concordia*, *Ostreococcus tauri*, *Micromonas commode; Choricystis* sp. *Nannochloris* sp., and *Platymonas* sp.), haptophytes (*Emiliania huxley* and *Pavlova lutheri* (non-calcifying), and dinoflagellates (*Thoracosphaera heimii*) (8, 22). On the cyanobacterial side, Si accumulation has been reported in marine *Synechococcus* strains representing four major phylogenetic clusters (4, 5). Altogether, these findings lead to the question of whether all non-siliceous phytoplankton are able to accumulate bSi. Our study reveals that brackish picocyanobacteria belonging to the Syn/Pro clade accumulate cellular Si, with quotas similar to the ones observed in the marine strains, which suggests that Si accumulation could be a common feature of this clade. Notably, we observed a lack of Si accumulation in two well-studied model freshwater coccoid cyanobacteria (*Synechococcus elongatus* PCC 7942 and *Synechocystis* PCC 6803). This suggests that these two strains can be used as negative controls in studies evaluating, for instance, factors regulating Si accumulation in non-siliceous phytoplankton. This resembles observations in heterotrophic bacteria of the genus *Bacillus* known to accumulate cellular Si, where strains assigned to *B. cereus* show pronounced dSi uptake, while *B. subtilis* show no accumulation (73). Our current findings thus indicate that (i) Si accumulation would not be a common feature of populations traditionally ascribed to the genus *Synechococcus* and (ii) not all phytoplankton without an obligate Si requirement accumulate this element.

### Si accumulation variability between strains

The Si content in natural populations from oligotrophic oceans can vary by at least an order of magnitude among picocyanobacterial cells from the same environmental sample. This goes in line with laboratory studies in which Si accumulation significantly varies between marine *Synechococcus* strains (4, 5, 11, 74). This could be explained by the fact that natural marine populations of *Synechococcus* consist of multiple coexisting ecotypes, which are genetically closely related but physiologically distinct (75, 76). The Baltic Sea picocyanobacterial community is also highly diverse with multiple coexisting ecotypes (38, 39, 77). Similarly, different bSi quotas were observed between the brackish picocyanobacterial strains in our study, which happen to be genetically closely related but different in terms of their physiology (38, 39). In the present study, differences in the Si content were not attributed to pigment types (e.g., phycocyanin-rich vs phycoerythrin-rich strains) or morphological characteristics of the strains (cell size, biovolume, or dry weight). Differences in Si quotas could not be attributed to the dSi conditions present when strains were isolated. BA and KAC strains were isolated from the Baltic Proper in late winter–spring when dSi concentrations were quite similar (ranging from 12 to 14 µM) (39, 78). As suggested for marine strains, different mechanisms for Si uptake (i.e., diffusion and a potential active transport mediated by phosphate transporters [5]), the chemical form of intracellular Si (e.g., soluble and insoluble internal Si pools), single-cell variability within populations, and other unknown physiological or environmental factors are likely to contribute to the variability in cellular Si quotas observed in the brackish strains.

Contrary to previous reports in marine clones (5), the highest Si quotas were observed in cells during the mid-exponential phase rather than the late-exponential or stationary phases. This observation could be explained by several factors. First, cells are likely larger during active growth in the mid-exponential phase compared to the late-exponential or stationary phases, enabling them to accumulate more Si. Second, during the exponential phase, cells are actively dividing, and it is possible that silica uptake temporarily increases as cells prepare for division and form new cell walls. Whether the increased Si quotas during this phase reflect a direct demand for silica or are a side effect of active growth, with potential biogeochemical consequences, remains unsolved. Third, actively growing cells may produce more extracellular polymeric substances (EPS), which could potentially bind additional Si. EPS production is species- and strain-specific and varies with growth conditions such as nutrient concentrations, salinity, and light and can also be associated with stress responses. EPS play a significant role in calcium precipitation, partly by providing nucleation sites for calcium carbonate. Similarly, EPS secreted by picocyanobacteria could serve as templates for Mg silicate formation, as observed in microbial mats (79).

Our analysis suggested a link between phylogeny and cellular Si accumulation, in which picocyanobacteria belonging to the Syn/Pro clade would accumulate Si, but earlier branching groups would not. This potential link between the phylogeny and patterns of silicification paves the way for future research to explore how evolutionary relationships influence silica utilization across diverse microbial communities and environmental conditions. Brackish and freshwater picocyanobacteria are represented by a mix of strains assigned to *Cyanobium* spp., *Synechocystis* spp., and *Synechococcus* spp. (80). Despite not belonging to the Syn/Pro clade, *Synechococcus elongatus is* often considered a model for freshwater *Synechococcus*. Recent comparative analyses reveal that the genomic capabilities for cellular processes vary significantly between freshwater picocyanobacteria belonging to the Syn/Pro clade and *Synechococcus elongatus* (81). Such genetic and metabolic differences likely account for the differences in Si accumulation observed between marine and brackish strains (belonging to the Syn/Pro clade) and *Synechococcus elongatus PCC 7942 and Synechocystis* PCC 6803 (two more divergent taxa). We consider that freshwater picocyanobacteria associated with the Syn/Pro clade could also accumulate Si in similar quotas to the ones found in the marine and brackish counterparts. Lastly, picocyanobacterial influence on Si cycling could be larger if *Prochlorococcus* also accumulates Si (4). The morphological similarities between *Synechococcus* and *Prochlorococcus* and its phylogenetic placement within the Syn/Pro clade allow us to propose that *Prochlorococcus* is also likely to accumulate Si. The cell abundances and ecological importance of both freshwater picocyanobacterial and *Prochlorococcus* call for further investigation of Si accumulation to elucidate their potential role in the global Si cycling.

### Mechanisms of biosilicification in picocyanobacteria

Acquisition of dSi is a key aspect of bSi formation, and biosilicifying organisms typically use specific SIT transporters that enable Si uptake from the environment (12, 13). In addition to SIT-Ls, we searched for the presence of *Lsi2* genes as they code for transmembrane transports mediating Si efflux in plant cells (72), show sequence orthology and strong similarity in function with the bacterial *ArsB* genes encoding arsenate transporter ArsB, and were recently suggested to be involved in Si uptake in sponges (82). Our finding of SIT-Ls in newly deposited genomes of cyanobacteria and heterotrophic bacteria expands the knowledge regarding their distribution in bacteria. In the same way, our searches uncovered for the first time *Lsi2* genes in strains of *Synechococcus elongatus*. Given the pronounced spatiotemporal differences in the distribution of distinct picocyanobacterial species and ecotypes, it will be intriguing to uncover the advantages conveyed by these dSi transporters to particular strains at precise environmental conditions and if related to growth or rather bring about improved survival during periods of challenging nutritional conditions.

The second step in biosilicification involves proteins involved in bSi deposition. Although the chemical form of bSi in picocyanobacteria is largely unresolved, studies in marine *Synechococcus* described one major water-soluble pool containing unpolymerized dSi and one water-insoluble silicon pool (5). In addition, spectroscopic analysis of dried *Synechococcus* cells showed the presence of a hydrated siliceous gel network, suggesting that at least a portion of the incorporated dSi molecules is polymerized (23). Our bioinformatic searches in the genomes of picocyanobacteria for proteins involved in Si deposition described in diatoms (e.g., silaffins, silacidins, and cingulins) (20) and sponges (e.g., silicatein and glassins) (1, 15) revealed no homologs. Likewise, the lack of differential gene expression in our picocyanobacterial cultures with and without dSi could suggest that polymerization in picocyanobacteria is spontaneous rather than mediated by transcriptional regulation of specific proteins. Knowledge of the chemical forms of bSi and the proteins involved in silicification (and the respective conserved domains) is far from complete even in known silicifiers (13), which certainly hampers attempts to identify picocyanobacterial counterparts.

### Technical and ecological effects of pH on bSi

The bulk alkaline-based digestion method applied to cells/particles collected on filters typically used to analyze cellular Si content (5, 8, 83, 84) does not differentiate biologically produced forms of Si from inorganic Si precipitation forms. The dSi can precipitate in the form of inorganic silicate phases at a pH higher than 8.5 (24), which can easily be reached in batch cultures of phytoplankton due to the uptake of inorganic carbon during photosynthesis. While this effect was tested early on in eukaryotic phytoplankton with no obligate requirement for dSi, like *Phaeodactylum tricornutum* (diatom) and *Platymonas* sp. (chlorophyte) (24), few if any, follow-up studies of this phenomenon are reported in the literature, and the effect has not been studied in cyanobacteria. Our experiments with picocyanobacteria showed that the removal of dSi when pH surpasses 8.5 comprises at least two components: a biologically controlled process (the biological uptake) combined with a biologically induced process (inorganic dSi precipitation when picocyanobacteria alter the pH). The comparison of assays under controlled pH (pH <8.5; Hepes buffer or bubbling) without control (pH 8.5–10) allowed us to determine that biologically induced precipitation can influence the cellular Si quotas up to 85%. This strongly reinforces the importance of pH control in laboratory experiments.

Beyond the importance of elevated pH on biologically induced dSi precipitation in laboratory experiments, we note that this process can have important ecological implications in natural systems, particularly in coastal and inland waters affected by phytoplankton blooms. When blooming, phytoplankton consumes large amounts of inorganic carbon, and pH values can frequently surpass 8.5 and reach values up to 10 (85). Biologically induced precipitation caused by elevated pH can significantly reduce the availability of dSi for Si-dependent groups like diatoms and thus hamper their ability to grow. In line with this, laboratory and field studies have demonstrated that pH conditions commonly associated with blooms of the colony-forming cyanobacterium *Microcystis aeruginosa* (pH ≥9.2) lead to decreased diatom growth rates, abundance, and silica deposition (86). Furthermore, competition experiments between *Phaeodactylum* (a diatom with no obligate Si requirement) and *Thalassiosira* (a diatom with an obligate Si requirement) support this pattern. Under conditions of high biomass, where sepiolite precipitation may occur, the Si-independent diatom is favored, further highlighting the competitive disadvantage faced by Si-dependent diatoms in such conditions (87). Moreover, diatom uptake of dSi is also influenced by pH in the range from 8.0 to 9.6 through its effect on the chemical form of Si (88). Three out of four investigated diatom species assimilated Si in the form of Si(OH)_4_, which is the dominant form at pH 8. However, as pH increases to 9.6, the proportion of Si in the form of SiO(OH)_3_^–^ increases substantially (effectively removing the monomer from the pool available for diatom uptake), leading to lowered Si uptake (88). Collectively, these findings imply double processes leading to reduced bioavailability of dSi at high pH, from changes in both the monomeric/ionic forms and precipitation. We thus argue that peaks in pH, driven by phytoplankton blooms (85), can induce largely unexplored feedback loops influencing phytoplankton dynamics and biogeochemistry. Studies during periods of elevated pH will be crucial for quantifying the extent of dSi precipitation (or other mineral precipitates) in highly productive coastal and freshwater systems.

Lastly, understanding how picocyanobacteria interact with silica provides valuable insights into both modern biogeochemical cycles and ancient sedimentary processes, such as stromatolite formation—layered sedimentary structures created by microbial communities, primarily cyanobacteria. This knowledge can refine the interpretations of the rock record, highlighting the significant role of biological activity in shaping sedimentary processes over geological time.

## Data Availability

Sequences generated in this study are available in GenBank under the accession numbers PP034391 to PP034406.

## SUPPLEMENTAL MATERIAL

Supplemental figures aem.02527-24-s0001.docx Figures S1 to S7.

Supplemental tables aem.02527-24-s0002.xlsx Tables S1 to S4.

ASM does not own the copyrights to Supplemental Material that may be linked to, or accessed through, an article. The authors have granted ASM a non-exclusive, world-wide license to publish the Supplemental Material files. Please contact the corresponding author directly for reuse.
