# Supplementary material for: The evaluation of biogenic silica in brackish and freshwater strains reveals links between phylogeny and silica accumulation in picocyanobacteria
Source: Appl Environ Microbiol. 2025 Mar 27;91(4):e02527-24. doi: 10.1128/aem.02527-24 (PMC12016540; doi:10.1128/aem.02527-24)
Supplement: Supplemental figures — Figures S1 to S7. [file aem.02527-24-s0001.docx]

**Supplementary material**

**The evaluation of biogenic silica in brackish and freshwater strains reveals links between phylogeny and silica accumulation in picocyanobacteria**

Anabella Aguilera^1,2*^, Daniel Lundin^1^, Evangelia Charalampous^1^, Yelena Churakova^1^, Christian Tellgren-Roth^3^, Sylwia Śliwińska-Wilczewska^4,5^, Daniel J. Conley^6^, Hanna Farnelid^1^, Jarone Pinhassi^1^

^1^Department of Biology and Environmental Science, Centre for Ecology and Evolution in Microbial Model Systems (EEMiS), Linnaeus University, Kalmar, Sweden

^2^ Science for Life Laboratory, Lund University, Lund, Sweden (present address)

^3^ Science for Life Laboratory, Department of Immunology, Genetics and Pathology, Uppsala University, Uppsala, Sweden

^4^Mount Allison University, Sackville, NB, Canada

^5^Laboratory of Marine Plant Ecophysiology, Institute of Oceanography, University of Gdansk, Gdynia, Poland

^6^Department of Geology, Lund University, Lund, Sweden

* Corresponding author: anabella.aguilera@scilifelab.se


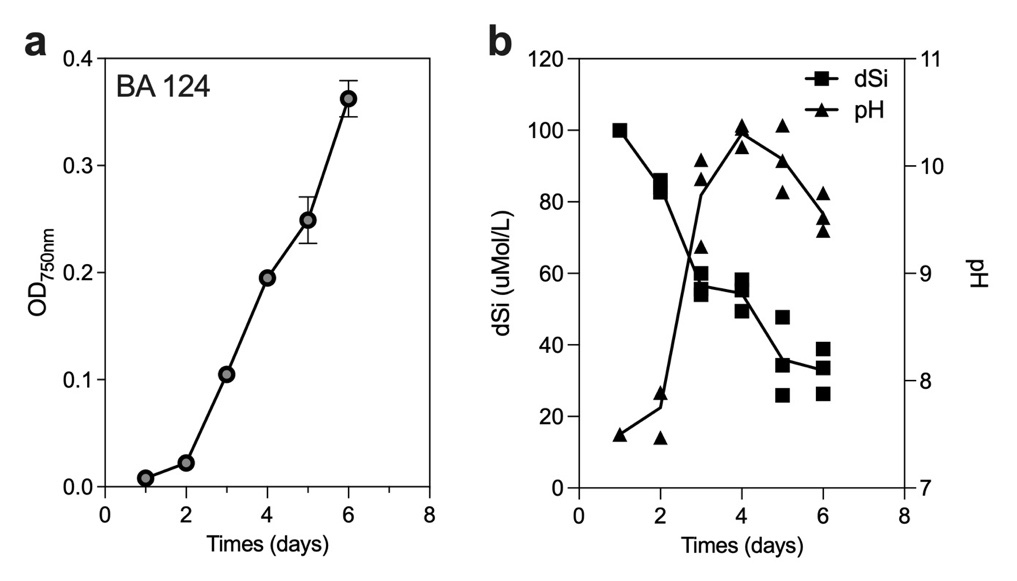


**Supplementary Figure 1.** Growth curve (a), and evolution of pH and dSi concentration (b) in batch culture with strain BA 124.


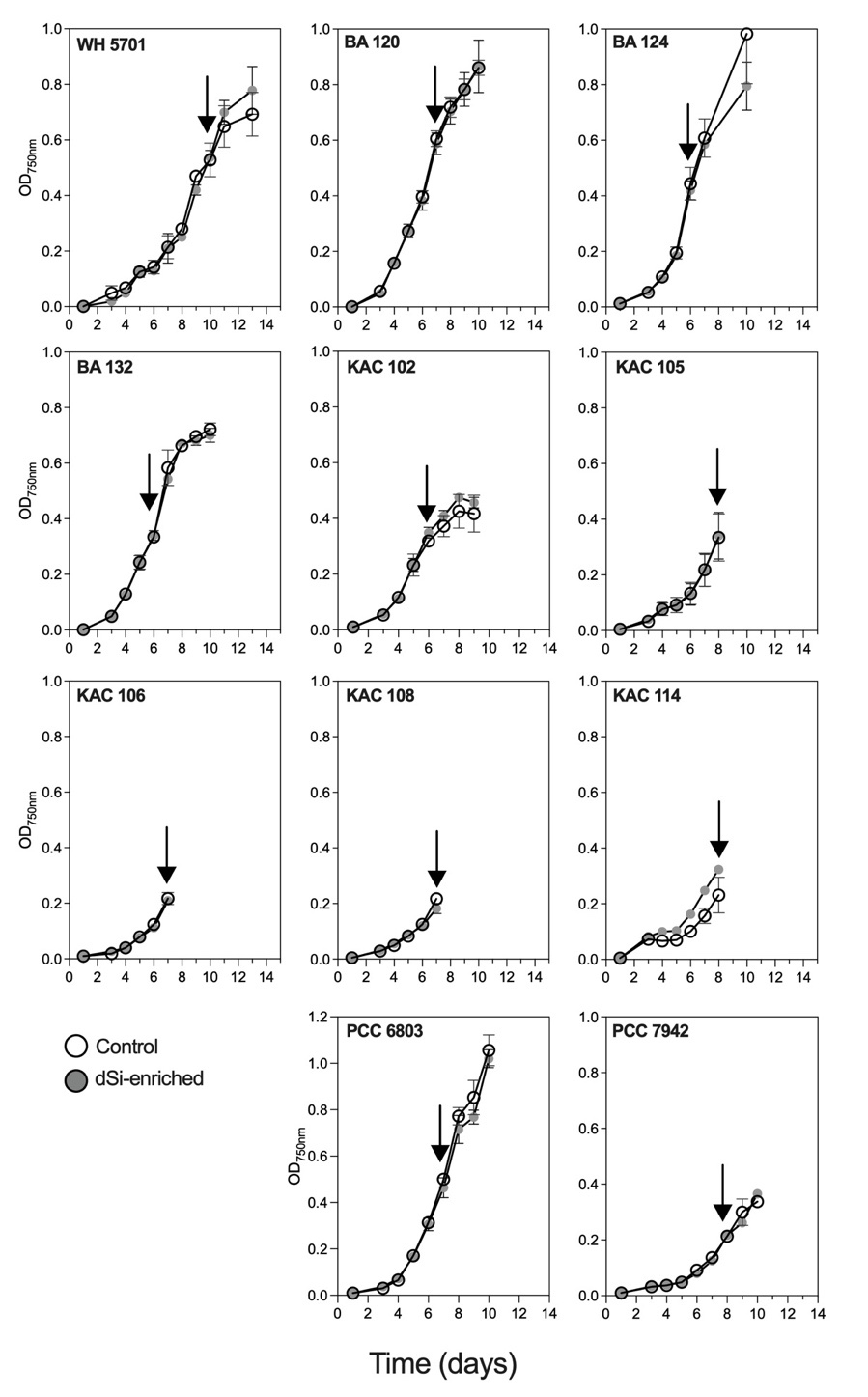


**Supplementary Figure 2.** Growth curves of marine, brackish, and freshwater picocyanobacterial strains cultured in control conditions (without added dSi) and in dSi-enriched media (+100 μM). The arrows indicate the time point at which samples were collected for bSi analysis.

**
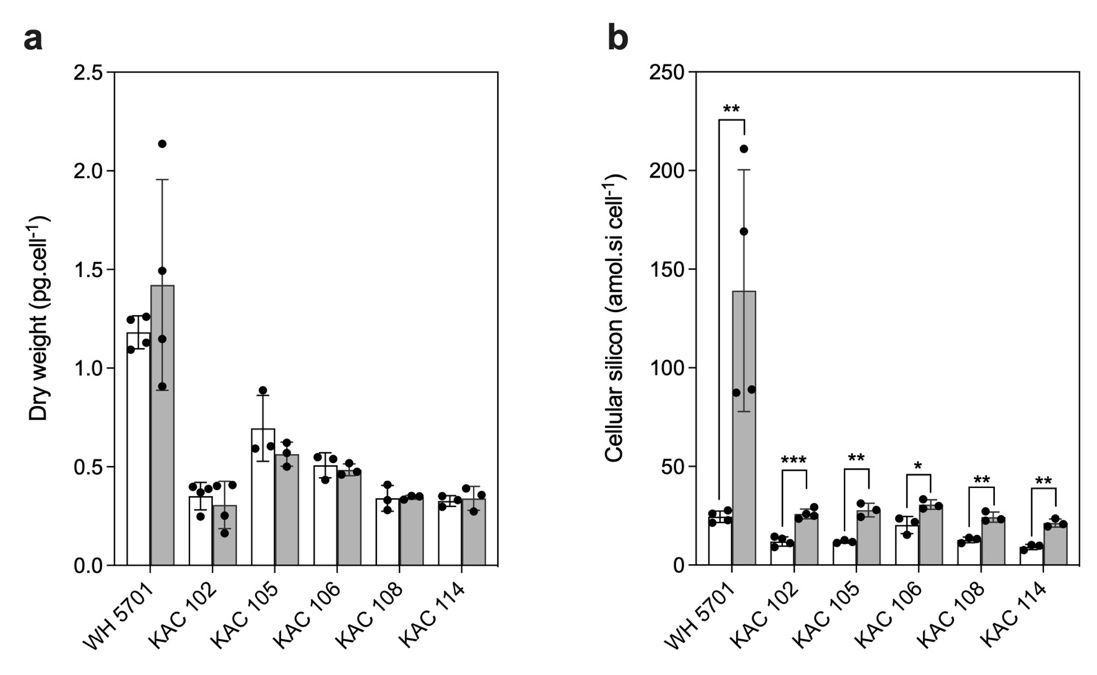
**

**Supplementary Figure 3.** Dry weight (a) and cellular silicon (b) in marine and brackish picocyanobacterial strains cultured in control conditions (without added dSi) and in dSi-enriched media (+100 μM). Asterisk(s) (*) indicate significant p values (one-way ANOVA, * p ≤ 0.05, ** p ≤ 0.01, *** p ≤ 0.001).


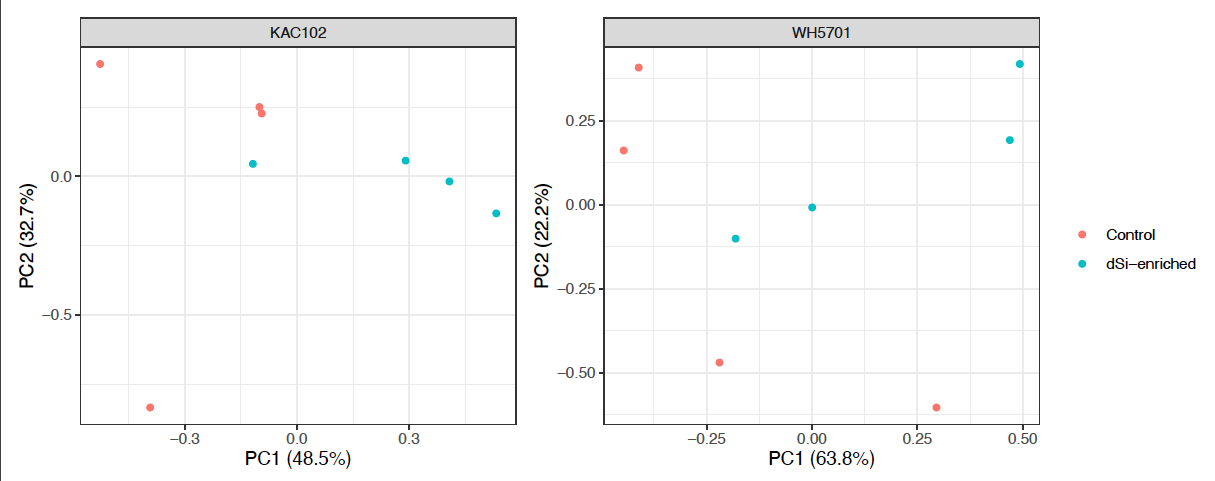


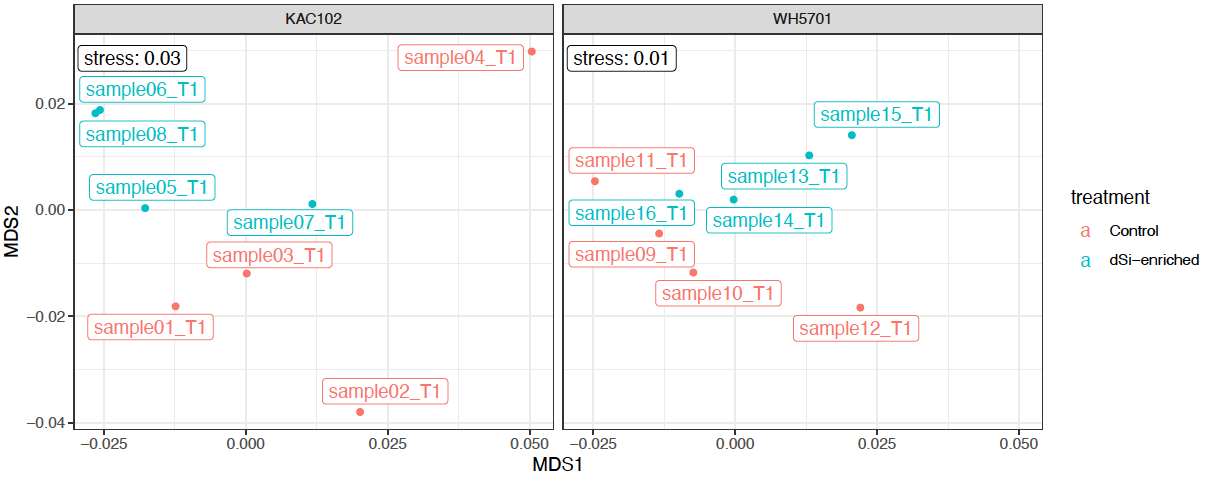


**Supplementary Figure 4:** Non-metric multidimensional scaling (b) and principal component analysis (b) of transcriptomes for the dSi-treated samples (blue) and control samples (red) for brackish strain KAC 102 and marine strain WH 5701.


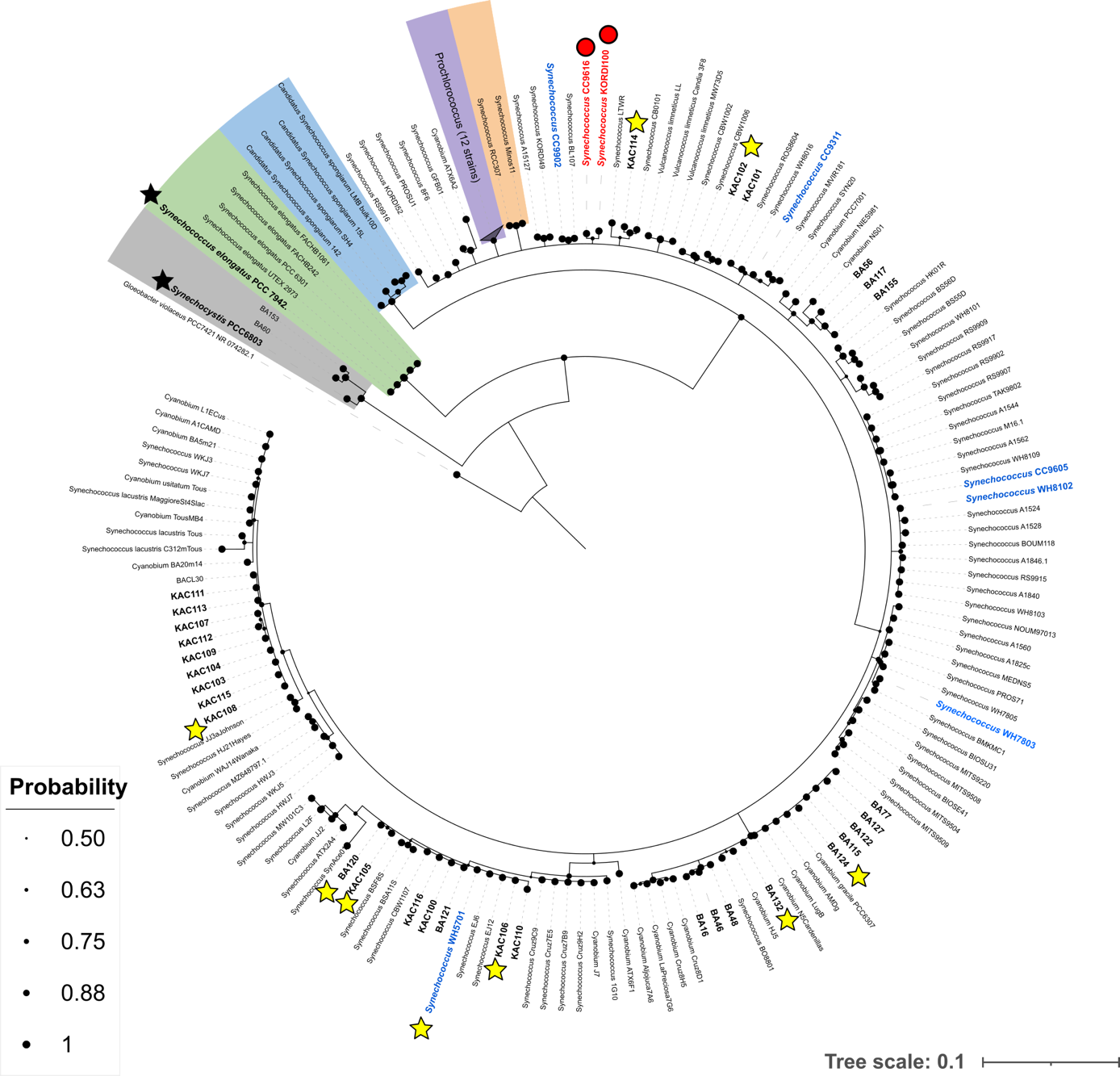


**Supplementary Figure 5: Phylogenetic position of picocyanobacterial strains used in this study and other strains shown to accumulate Si using Bayesian Inference.** Red circles indicate strains harboring SIT-Ls (1), and strains in blue were previously shown to accumulate Si (2). Stars indicate strains tested for Si accumulation in this study: yellow stars indicate strains that accumulated Si and black stars indicate those that did not accumulate Si. All BA strains and KAC strains (in bold) were screened for SIT-Ls. Phylogenies were constructed using MrBayes v3.2.7a (3) under GTR+I+gamma model (lset nst=6 rates=invgamma).


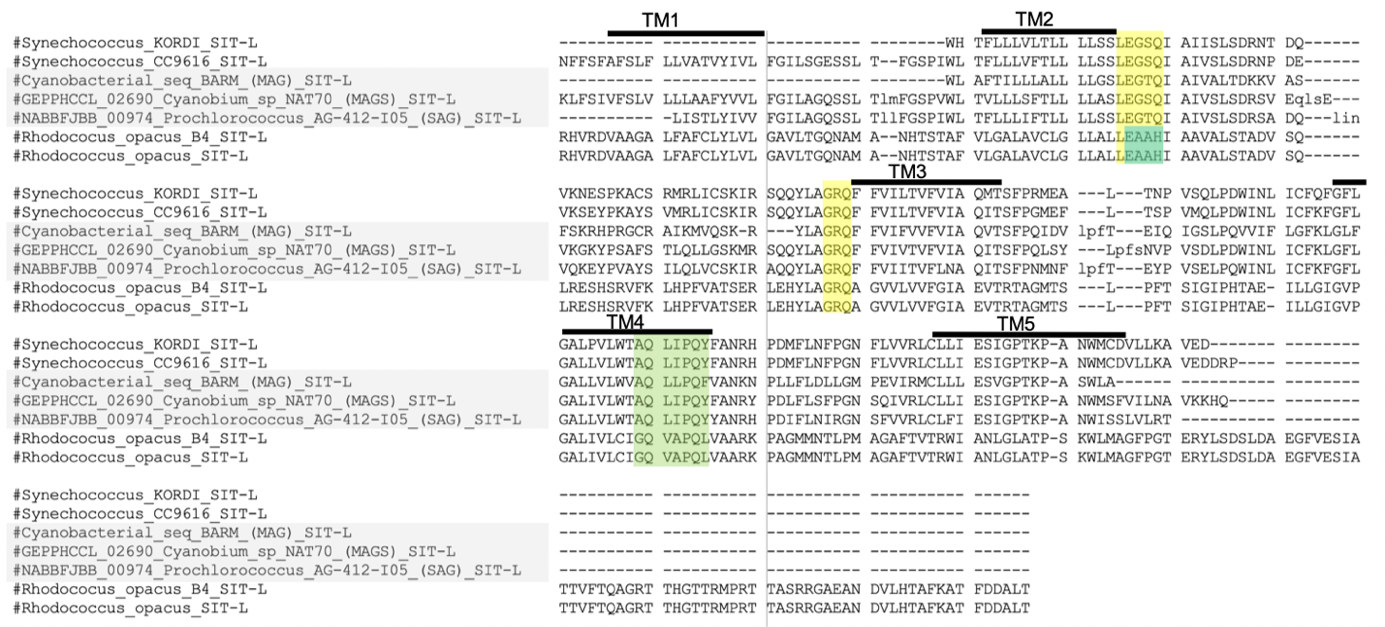


**Supplementary Figure 6. Full alignment of SIT-Ls from reference bacterial sequences and sequences affiliated with picocyanobacteria found in this study, highlighting transmembrane domains and conserved features.** Transmembrane region annotations are based on matching the five transmembrane domains predicted for SIT-Ls (1, 4). Conserved EGXQ and GRQ motifs are highlighted in yellow and conserved xQxxxQ motifs are indicated in green. The grey boxes denote novel picocyanobacteria SIT-Ls found in this study.


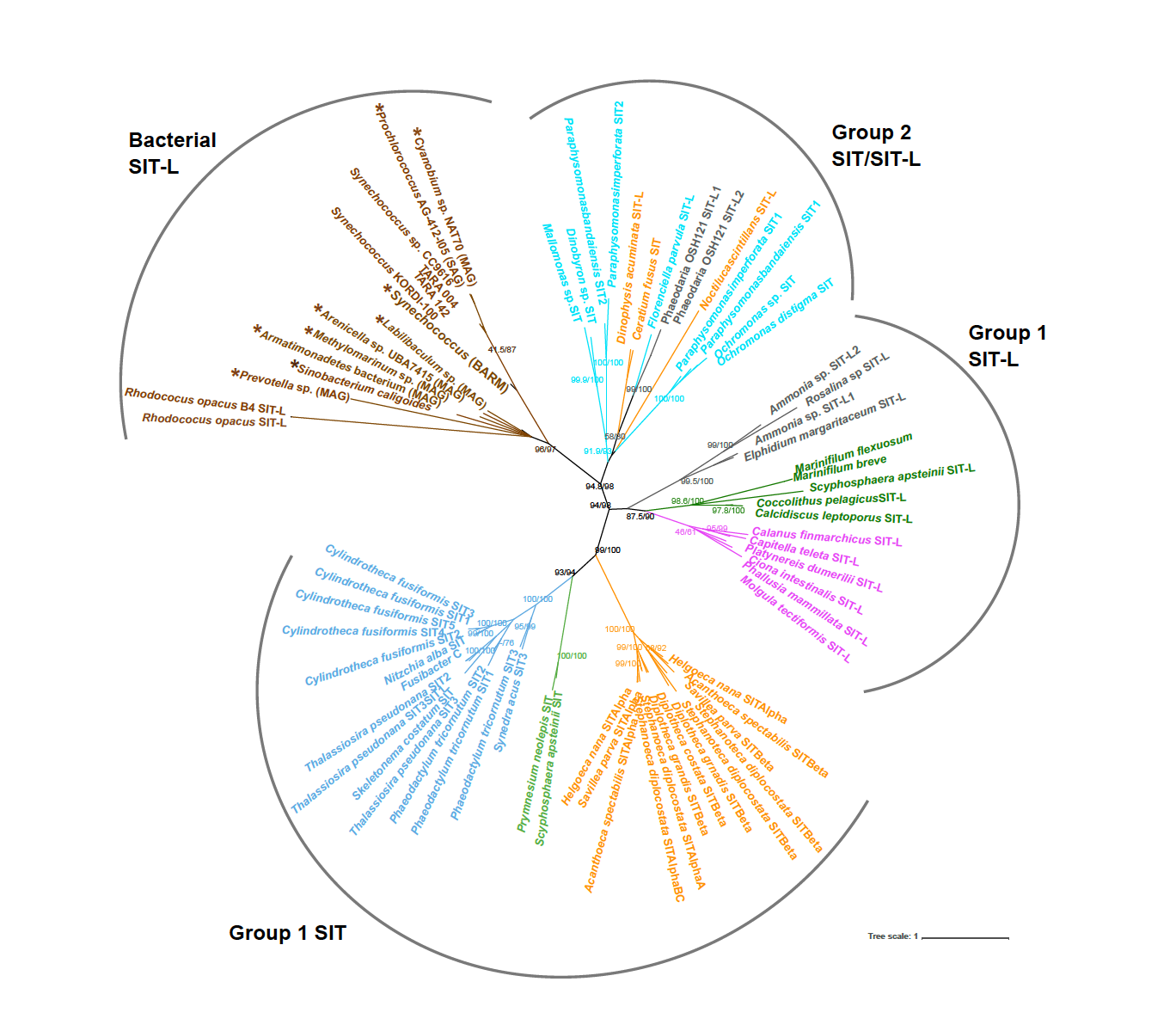


**Supplementary Figure 7. Phylogenetic tree showing the phylogenetic placement of bacterial SIT-L sequences found in this study.** Sequences found in the study (n=9, indicated with asterisks) were placed onto a reference tree previously published by (1) via a reference alignment using phylogenetic placement. Numbers at nodes are a percentage of 100 bootstrap replicates in the format of support value. The scale bar indicates average number of amino acid substitutions per site.

**References**

1. Marron AO, Ratcliffe S, Wheeler GL, Goldstein RE, King N, Not F, de Vargas C, Richter DJ. 2016. The Evolution of Silicon Transport in Eukaryotes. Mol Biol Evol 33:3226–3248.

2. Brzezinski MA, Krause JW, Baines SB, Collier JL, Ohnemus DC. 2017. Patterns and regulation of silicon accumulation in Synechococcus spp. J Phycol.

3. Ronquist F, Teslenko M, van der Mark P, Ayres DL, Darling A, Höhna S, Larget B, Liu L, Suchard MA, Huelsenbeck JP. 2012. MrBayes 3.2: efficient bayesian phylogenetic inference and model choice across a large model space. Syst Biol 61:539–542.

4. Durak GM, Taylor AR, Walker CE, Probert I, de Vargas C, Audic S, Schroeder D, Brownlee C, Wheeler GL. 2016. A role for diatom-like silicon transporters in calcifying coccolithophores. 1. Nat Commun 7:10543.
